# A systematic evaluation of nucleotide properties for CRISPR sgRNA design

**DOI:** 10.1186/s12859-017-1697-6

**Published:** 2017-06-06

**Authors:** Pei Fen Kuan, Scott Powers, Shuyao He, Kaiqiao Li, Xiaoyu Zhao, Bo Huang

**Affiliations:** 1 Department of Applied Mathematics and Statistics, Stony Brook University, 100 Nicolls Road, Stony Brook, 11794 USA; 2 Department of Pathology, Stony Brook University, 100 Nicolls Road, Stony Brook, 11794 USA; 3 Oncology Business Unit, Pfizer Inc., 558 Eastern Point Rd, Groton, 06340 USA

**Keywords:** CRISPR, Machine learning, Predictive modeling, Thermodynamics

## Abstract

**Background:**

CRISPR is a versatile gene editing tool which has revolutionized genetic research in the past few years. Optimizing sgRNA design to improve the efficiency of target/DNA cleavage is critical to ensure the success of CRISPR screens.

**Results:**

By borrowing knowledge from oligonucleotide design and nucleosome occupancy models, we systematically evaluated candidate features computed from a number of nucleic acid, thermodynamic and secondary structure models on real CRISPR datasets. Our results showed that taking into account position-dependent dinucleotide features improved the design of effective sgRNAs with area under the receiver operating characteristic curve (AUC) >0.8, and the inclusion of additional features offered marginal improvement (∼2% increase in AUC).

**Conclusion:**

Using a machine-learning approach, we proposed an accurate prediction model for sgRNA design efficiency. An R package predictSGRNA implementing the predictive model is available at http://www.ams.sunysb.edu/~pfkuan/softwares.html#predictsgrna.

**Electronic supplementary material:**

The online version of this article (doi:10.1186/s12859-017-1697-6) contains supplementary material, which is available to authorized users.

## Background

Clustered Regularly Interspaced Short Palindromic Repeats (CRISPR)/Cas system is a heritable and adaptive prokaryotic immune system that protects cells by destroying foreign genetic elements [1]. Over the past few years, CRISPR has emerged as a powerful gene editing technology [2, 3]. CRISPR consists of a single guide RNA (sgRNA) and an enzyme called Cas9. The sgRNA is composed of a short synthetic RNA (approximately 20 base pairs (bp), known as spacer target) located within a N-bp scaffold. The spacer target is designed to bind to a specific sequence in the genome, whereas the Cas9 protein acts as a biomolecular scissor. This system has proven to be a powerful tool for studying individual gene function and for genome engineering.

The design of sgRNA is an important aspect to ensure the success of CRISPR-Cas9 screens. It is desirable to design sgRNA libraries which have maximum on-target and minimum off-target effects. The binding specificity of the sgRNA is determined by the 20 bp spacer target and a protospacer adjacent motif (PAM) sequence (generally NGG or NAG) on the genome. Once the sgRNA binds to the target sequence, the Cas9 nuclease cuts 3-bp upstream of the PAM sequence. Different groups have studied the sequence features of spacer target sites that predict sgRNA on-target efficiency [4–7]. In particular, [5] investigated the position-dependent sequence on sgRNA efficiency and whether these features could reproducibly predict sgRNA efficiency in several publicly available CRISPR datasets. They proposed a predictive model using the position-dependent mono-nucleotide composition across a 40 bp sequence encompassing 5’ flanking, spacer target and 3’ flanking region; and further demonstrated that their model performed better than the model of [4]. On the other hand, [6, 7] proposed a predictive model based on gradient-boosted regression trees using position-dependent and independent sequence properties, location of the sgRNA within the protein and melting temperatures.

Aspects of sgRNA design share similarities to oligonucleotide designs used for microarrays. In both cases, optimal oligonucleotide design aims to increase binding sensitivity and specificity while minimizing off target hybridization. A position dependent sequence bias has been observed in the design of oligonucleotides in Affymetrix microarrays [8], whereas in our earlier work [9] we showed that the thermodynamic and secondary features of the oligonucleotides affect the hybridization intensities in Nimblegen arrays. In addition, [6, 7] investigated position dependent and independent features, position of the guide within the genes, interaction with the PAM sequence and melting temperatures, and showed that these features improved the prediction model in CRISPR/Cas9 screens; whereas microhomology features did not improve the prediction. In this paper, we computed a comprehensive list of features of the target sequence from a number of nucleic acid, thermodynamic, and secondary structure models by adopting some ideas of microarray designs. In a similar manner as [6, 7], we systematically characterized the effect of these features on the efficiency of sgRNA design, and seek to understand if the inclusion of these features improves the design of effective sgRNAs in CRISPR/Cas9 knockout screens.

## Methods

We used the sets of efficient and inefficient sgRNAs from the CRISPR/Cas9 screens of [10] and [11] compiled by [5]. The first dataset consists of 731 efficient and 438 inefficient sgRNAs targeting ribosomal genes [10], the second dataset consists of 671 efficient and 237 inefficient sgRNAs targeting non-ribosomal genes [10] and the third dataset consists of 830 efficient and 234 inefficient sgRNAs targeting essential genes in mouse embryonic stem cell (mESC) line, JM8 [11]. The procedures for identifying efficient and inefficient sgRNAs were used exactly as described in [5]. Spacer lengths in the reported studies were 20 bp [10] and 19 bp [11]. Using these sets of sgRNAs, we computed primary sequence, thermodynamic, and secondary structures as candidate features. Further details are provided below.

### DNA sequence candidate features

#### Position-dependent nucleotide composition

Similar to [5], we created vectors of position-dependent mono-nucleotide composition (PD Mono) for the 40 bp long sequences comprised of the spacer targets, and 5’ and 3’ flanking regions. In addition, we extracted position-dependent dinucleotide composition (PD Dinuc) for these 40 bp sequences and computed the single and dinucleotide frequencies (Freq) for the spacer target. Since positions 32 and 33 were part of the PAM sequence (GG), they were excluded from the analysis.

#### Thermodynamics and secondary structure properties of [9] (Thermo)

Motivated by our earlier work which studied the relationship between oligonucleotide properties and hybridization signal intensities in microarray design [9], we computed the thermodynamic properties: melting temperature (*T*_*m*_), GC content, entropy change (*Δ*S), enthalpy change (*Δ*H), free energy change (*Δ*G); and secondary structures: longest polyN, repetitive sequence (repeat), length of a potential stem-loop (LSL) and minimum energy folding (MEF). *T*_*m*_ was computed according the formula

$$ \kern0.9em {T}_m=81.5+16.6\left(\underset{10}{ \log}\left(\left[ N{a}^{+}\right]\right)\right)+0.41\ast \left(\%\mathrm{GC}\right)-600/ L $$

where [*Na* ^+^] was assumed to be 0.2M [12]. *Δ*G, *Δ*H and *Δ*G were calculated by summing the respective entropy, enthalpy and free energy parameters of each dinucleotide, including the initiation parameters and penalty for self complementary duplexes according to the position-dependent nearest neighbor approach as described [13]. These parameters were provided in Tables 1 and 2 of [13]. MEF was computed using the hybrid-ss-min program in OligoArrayAux package, whereas LSL was computed using the palindrome function in the EMBOSS package. Longest polyN and repeat were calculated as previously described [9]. These properties were computed for the spacer target sequence.

#### DNA secondary structures based on dinucleotide and tetra nucleotide properties of [14] and [15] (Packer)

Following a previously described approach [16], we computed the minimum, maximum and average values of both the tetranucleotide energy and flexibility scores as described [15]. These scores were given in Tables 3 and 4 of [15]. In addition, we computed the minimum, maximum and average values of the dinucleotide roll, twist, slide and shift scores as described [14]. The dinucleotide values of these properties were given in Tables 1, 2 and 3 of [14]. These scores were representations of the three-dimensional DNA structure and anisotropic flexibility [14]. Similar to above, we computed these properties for the spacer target sequence.

#### Physiochemical properties of [17] (PhyChem)

We adapted the approach described by [17] which was developed for predicting nucleosome occupancy and computed the 12 physiochemical properties (A-pillicity, base-stacking, B-DNA twist, bendability, DNA bending stiffness, DNA denaturation, duplex disrupt energy, duplex free energy, propeller twist, protein deformation, protein-DNA twist and Z-DNA). For each property, we computed the minimum, maximum and average dinucleotide scores for the spacer target sequence. The dinucleotide values of the 12 physicochemical properties were given in Table 1 of [17].

#### Pseudo k-tuple nucleotide composition of [18] (PseKNC)

The PseKNC model was also originally developed for predicting nucleosome occupancy by taking into account global sequence-order effects. PseKNC represents the DNA sequence as vectors $\left [\frac {f_{1}}{d},\hdots,\frac {f_{4^{k}}}{d},\frac {w\theta _{1}}{d},\hdots,\frac {w\theta _{\lambda }}{d}\right ]^{T}$ where $d={\sum \nolimits }_{j=1}^{4^{k}}f_{j}+w{\sum \nolimits }_{j=1}^{\lambda } \theta _{j}, f_{j}$’s are the k-tuple nucleotide frequencies and

$$\theta_{j}=\frac{1}{m(L-j-1)}\sum\limits_{s=1}^{L-j-1}\sum\limits_{t=1}^{m}\left[P_{t}(r_{s}r_{s+1})-P_{t}\left(r_{s+j}r_{s+j+1}\right)\right]^{2} $$

*m* is the number of local DNA properties considered, *P*_*t*_(*r*_*s*_ *r*_*s*+1_) and *P*_*t*_(*r*_*s*+*j*_ *r*_*s*+*j*+1_) are the score of the *t*-th DNA local structural property for dinucleotide *r*_*s*_ *r*_*s*+1_ and *r*_*s*+*j*_ *r*_*s*+*j*+1_ at position *s* and *s*+*j*, respectively. *λ* is the order of correlations along the DNA sequence and *w* is the weight factor. Our candidate *k*, *λ* and *w* took values of *k*=2,3,…,6, *λ*=1,2,…,15, and *w*=0,0.1,0.2,…,1. We considered the following strategy to choose the optimal parameters for the PseKNC model. A three way cross validation was performed on each dataset using elastic net [19]. The parameters corresponding to the PseKNC model with the largest average area under the receiver operating characteristic curve (AUC) were selected for subsequent analysis. Based on this criterion, we set *k*=2, *λ*=1 and *w*=0.5. Similar to [18], we considered *m*=6 DNA local structural properties which were divided into local translational (rise, slide and shift) and angular (twist, roll and tilt).

#### Optimal pairwise alignment (Align)

We computed the optimal global pairwise alignment scores between the seed region and scaffold using the Needleman-Wunsch algorithm [20] which served as a measure of the potential of the *k* PAM-proximal seed region of the spacer target to interact with the scaffold sequence. The seed region was defined as the immediate *k* nucleotides next to the PAM sequence. We considered *k*=5,…,*L*, where *L* is the length of spacer target.

## Results and discussion

**Fig. 1:**
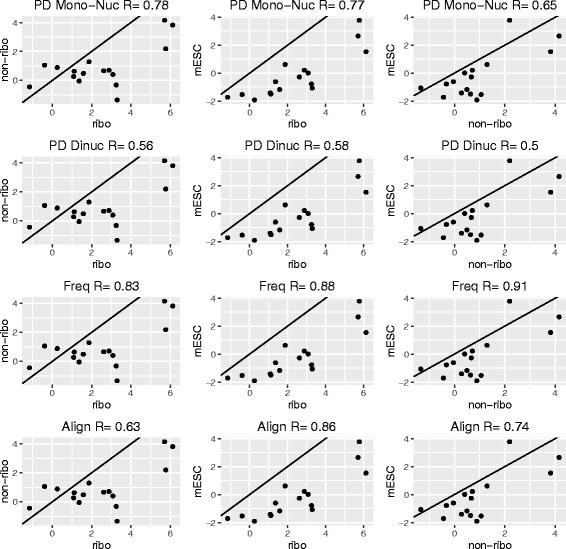
Pairwise correlation plot for each class of features. *Left* column is the pairwise correlation plot between ribosomal and non-ribosomal genes from [10]. *Middle* column is the pairwise correlation plots between ribosomal genes from [10] and mESC essential genes from [11]. *Right* column is the pairwise correlation plots between non-ribosomal genes from [10] and mESC essential genes from [11]. Each point is a feature

**Fig. 2:**
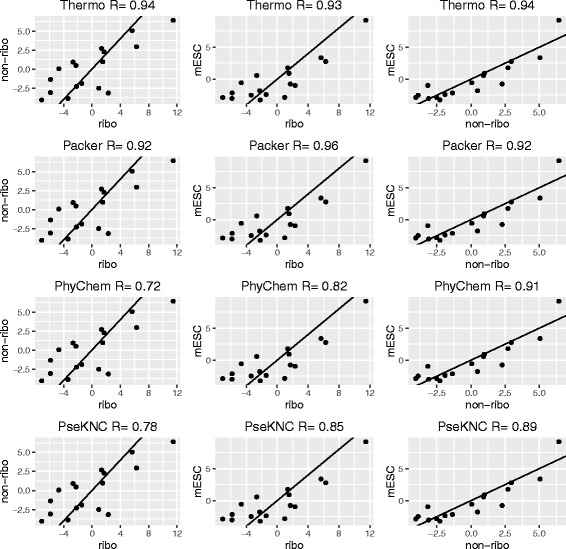
Pairwise correlation plot for each class of features. *Left* column is the pairwise correlation plot between ribosomal and non-ribosomal genes from [10]. *Middle* column is the pairwise correlation plots between ribosomal genes from [10] and mESC essential genes from [11]. *Right* column is the pairwise correlation plots between non-ribosomal genes from [10] and mESC essential genes from [11]. Each point is a feature

For each dataset, we computed a score for every feature as a measure of strength of association with sgRNA efficiency. If the feature was a binary variable, a log odds ratio between efficient and inefficient sgRNAs was computed. If the feature was a continuous variable, two-sample t-statistic was computed. We divided the features into 8 classes (1) position-dependent mono-nucleotide (PD Mono), (2) position-dependent dinucleotide (PD Dinuc), (3) frequencies of mono and dinucleotides (Freq) (4) optimal pairwise alignment between spacer target and scaffold (Align) (5) thermodynamics and secondary structures of [9] (Thermo) (6) secondary structures of [14, 15] (Packer) (7) physiochemical properties (PhyChem) of [17] and (8) pseudo k-tuple nucleotide composition of [18] (PseKNC). We found that most of the features were consistently associated with sgRNA efficiency across datasets (Figs. 1 and 2).

### Candidate feature ranking

**Fig. 3:**
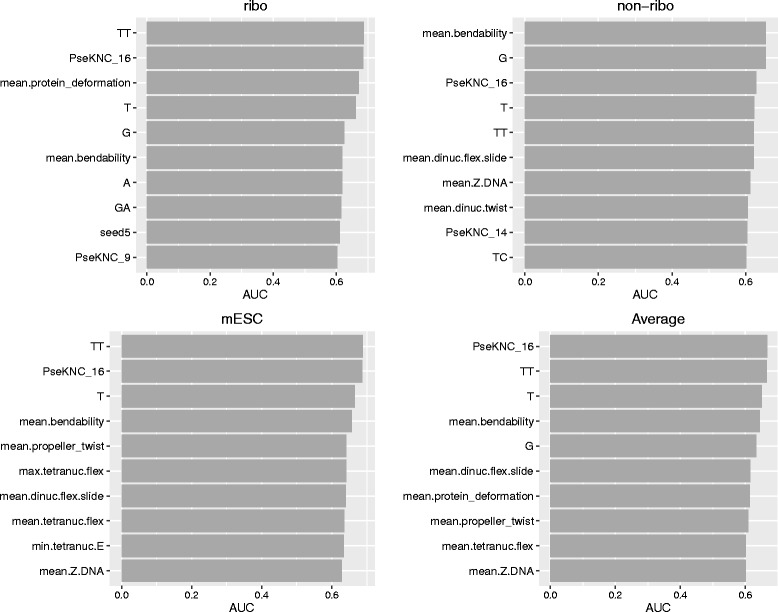
Top 10 most informative features ranked by AUC by dataset. The last panel is the ranking by average AUC aggregating the three datasets

To rank the contribution of each feature to the efficiency of sgRNA design, we fitted a logistic regression model within each dataset using the binary sgRNA efficiency indicator as the response and the features as predictors. The Bayesian Information Criterion (BIC) for the fitted model was computed. The features were ranked by the BIC scores and the top 10 most important features were shown in Additional file 1: Figure S1. The top ranked feature based on average BIC scores across the three datasets was the 16-th feature from PseKNC model. This feature is a function of TT dinucleotide frequency. In addition, we computed the area under receiver operating characteristic curves (AUCs) for continuous features. The top 10 features ranked by AUC were shown in Fig. 3, in which the 16-th feature from the PseKNC model was also ranked number one. The third measure we considered for feature ranking was the permutation based variable importance score from the random forest prediction algorithm. Random forest [21] is a non-parametric ensemble approach based on a large number of classification trees trained on bootstrap samples. The permutation based variable importance score of a feature is defined as the difference in prediction accuracy before and after permuting this feature, averaging over all trees. We used the unscaled version of variable importance score as recommended by [22, 23] to avoid bias due to number of trees grown. The top 10 features ranked by variable importance are shown in Additional file 1: Figure S2. Based on these results, the frequencies of T and TT had the strongest association with sgRNA efficiency, in which higher frequencies of T and TT were associated with decreased efficiency.

### Predictive modeling

To assess the contribution of the 8 different feature classes in prediction sgRNA efficiency, we formed all possible combinations of feature classes (${\sum \nolimits }_{i=1}^{8}{8\choose i}=255$ combinations). We adapted the strategy in [5] in constructing and evaluating the predictive model for sgRNA efficiency:

1. To evaluate intra-platform consistency within the same class of genes, we performed 3-way cross validation within dataset 1 (sgRNA targeting ribosomal genes) from [10]. We randomly split dataset 1 into 3 parts of equal sample size, trained the model on two parts (training set) and evaluated the performance of the resulting predictive model on the remaining part (test set). This process was repeated 3 times by leaving out a different test set, and results were averaged over 10 iterations of random sampling.
2. To evaluate intra-platform consistency across different classes of genes, the predictive algorithm was trained on dataset 1 (ribosomal genes) and tested on dataset 2 (non-ribosomal genes).
3. To evaluate inter-platform consistency, the predictive algorithm was trained on datasets 1 and 2 (ribosomal + non-ribosomal genes) from [10] and tested on dataset 3 (mESC essential genes) from [11].

The elastic net algorithm [19] was used in constructing the predictive model on the training set based on 10 fold cross-validation. Since the features we considered in this paper were functions of the nucleotide composition, they were correlated and the elastic net algorithm automatically selected non-redundant informative features. The objective function of elastic net consists of a loss function + penalty:

$$\min_{\mathbf{\beta}}||\mathbf{y}-\mathbf{X\beta}||^{2}+\lambda\left\{\alpha||\mathbf{\beta}||_{1}+(1-\alpha)||\mathbf{\beta}||^{2}\right\} $$

where $||\mathbf {\beta }||_{1}={\sum \nolimits }_{j=1}^{p}|\beta _{j}|$ and $||\mathbf {\beta }||^{2}={\sum \nolimits }_{j=1}^{p}\beta _{j}^{2}$.

We evaluated the performance on the test set in terms of AUC. The optimal cutpoints were determined by maximizing the Youden index(*J*) =Se+Sp −1, where Sensitivity(Se)$=\frac {TP}{TP+FN}$ and Specificity(Sp)$=\frac {TN}{TN+FP}$. The results were shown in Tables 1, 2 and 3. For each test set, we reported these performance measures for the predictive models constructed using each of the 8 feature classes, as well as the combinations of feature classes with the maximum AUC (Comb Feature). Across all comparisons, integrating multiple feature classes showed improvements in terms of AUC compared to position-dependent mono-nucleotide models (PD Mono) in [5]. Among the 8 individual feature classes, position-dependent dinucleotide models (PD Dinuc) consistently outperformed other feature classes in predicting sgRNA efficiency and were close to results from the combination of feature classes models in all 3 scenarios. A similar pattern was also observed in [6, 7], in which they showed that position dependent dinucleotide features yielded the largest average Gini importance among the set of features considered in their dataset [4, 7].

**Table 1:** AUC, Youden index (*J*), Sensitivity (Se) and Specificity (Sp) from the 3-way cross validation within dataset 1 (ribosomal genes)

Feature class	AUC	*J*	Se	Sp
PD Mono	0.826	0.535	0.855	0.680
PD Dinuc	0.848	0.575	0.788	0.787
Freq	0.778	0.441	0.677	0.764
Align	0.613	0.188	0.746	0.442
Thermo	0.525	0.086	0.812	0.273
Packer	0.601	0.186	0.634	0.551
PhyChem	0.722	0.380	0.711	0.669
PseKNC	0.731	0.376	0.683	0.693
Comb Feature	0.867	0.618	0.826	0.792

Comb Feature: PD Mono+PD Dinuc+*Freq*+*Thermo*+*Packer*+*PhyChem*+PseKNC. We reported the average performance from the 3-way cross validation over 10 iterations of random sampling

**Table 2:** AUC, Youden index (*J*), Sensitivity (Se) and Specificity (Sp) from intra-platform comparison (training set: ribosomal genes, test set: non-ribosomal genes)

Feature class	AUC	*J*	Se	Sp
PD Mono	0.785	0.443	0.717	0.726
PD Dinuc	0.792	0.478	0.765	0.713
Freq	0.700	0.332	0.779	0.553
Align	0.594	0.159	0.881	0.278
Thermo	0.616	0.222	0.639	0.580
Packer	0.637	0.207	0.431	0.776
PhyChem	0.659	0.241	0.633	0.608
PseKNC	0.647	0.243	0.694	0.549
Comb Feature	0.806	0.492	0.851	0.641

Comb Feature: PD Mono+PD Dinuc +*Thermo*+*Packer*+PhyChem

**Table 3:** AUC, Youden index (*J*), Sensitivity (Se) and Specificity (Sp) from inter-platform comparison (training set: ribosomal and non-ribosomal genes, test set: mESC essential genes)

Feature class	AUC	*J*	Se	Sp
PD Mono	0.797	0.486	0.751	0.735
PD Dinuc	0.832	0.544	0.792	0.752
Freq	0.751	0.382	0.716	0.667
Align	0.574	0.131	0.490	0.641
Thermo	0.641	0.261	0.817	0.444
Packer	0.667	0.241	0.514	0.726
PhyChem	0.726	0.351	0.718	0.632
PseKNC	0.733	0.370	0.660	0.709
Comb Feature	0.848	0.566	0.843	0.722
azimuth	0.795	0.463	0.857	0.607
sgRNA Scorer	0.669	0.288	0.548	0.739
azimuth (retrained)	0.833	0.543	0.787	0.756
sgRNA Scorer (retrained)	0.804	0.474	0.786	0.688

Comb Feature: PD Mono+PD Dinuc+*Freq*+*Align*+*Thermo*+*Packer*+*PhyChem*+

PseKNC. azimuth and sgRNA Scorer were the results based on the softwares by [7] and [27], respectively developed using different training datasets. azimuth (retrained) and sgRNA Scorer (retrained) were the results obtained by refitting the algorithms on the current training set (ribosomal and non-ribosomal genes)

We also compared the results using the random forest and boosted regression to construct the predictive model. Random forest [21] was implemented in the R package randomForest, whereas the boosted regression based on extensions to AdaBoost [24] and gradient boosted machine [25] was implemented in the R package gbm. The results were shown in Additional file 1: Tables S1, S2 and S3 (randomforest) and Additional file 1: Tables S4, S5 and S6 (gbm). These results were comparable to the results from elastic net.

**Fig. 4:**
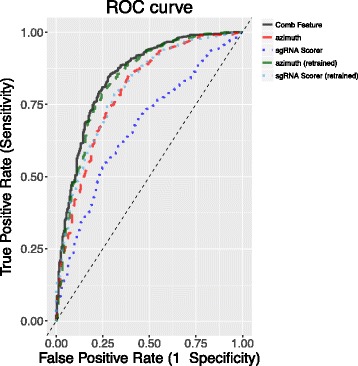
AUC curves for our proposed predictive model using combination features (Comb Feature), azimuth and sgRNA scorer. azimuth and sgRNA Scorer were the results based on the softwares by [7] and [27], respectively developed using different training datasets. azimuth (retrained) and sgRNA Scorer (retrained) were the results obtained by refitting the algorithms on the current training set (ribosomal and non-ribosomal genes)

Related work for predicting CRISPR/Cas9 guide efficiency based on nucleotide properties and melting temperatures includes azimuth [4, 6, 7], which constructed a predictive model based on gradient-boosted regression trees as described earlier. This method was recommended by [26] for in-vivo (U6) transcribed guides. In contrast, the sgRNA scorer of [27] was a predictive model based on the support vector machine (SVM) algorithm using position dependent mono-nucleotide on 5’ flanking (5 bp), spacer target and 3’ flanking (NGG + 5 bp) region. We included these two methods for comparison in Table 3 and Fig. 4. In this comparison, each method was trained on different datasets, but the performance was evaluated on the same test dataset generated by an independent research group, i.e., [11] dataset. The statistical significance for pairwise AUC comparisons was based on DeLong’s test [28]. Our proposed predictive algorithm achieved higher AUC compared to both azimuth and sgRNA scorer (*p*<0.001 in both cases). On the other hand, azimuth had better performance than sgRNA scorer (*p*<0.001). We have also implemented azimuth (based on continuous outcome gbm model) and sgRNA scorer (based on binary outcome SVM model) using the sequence features identified by [6, 7] and [27], respectively on the same training data (i.e., [10] ribosomal and non-ribosomal genes) (Table 3). As expected, the performance of sgRNA scorer was comparable to the model using position dependent mono-nucleotide (Table 3), whereas the performance of azimuth was comparable to the gbm results in Additional file 1: Table S15. Our proposed predictive algorithm achieved higher AUC compared to the refitted sgRNA scorer (*p*=0.048) and comparable performance to the refitted azimuth (*p*>0.1).

We also included comparison using a regression model based on (1) the average log2 fold change (12 cell doublings vs initial seeding states) of HL-60 and KBM-7 cell lines for [10] data and (2) the average log2 fold change (mESC vs plasmid control) of replicate 1 and replicate 2 of mouse ESC JM8 cell lines for [11] data. We compared the performance of the sequence properties in prediction in terms of AUC, Pearson correlation coefficient, Spearman rank correlation coefficient and mean squared error on the test data. The results were presented in Additional file 1: Tables S7, S8 and S9. In addition, similar to the binary outcome model as described above; position-dependent dinucleotide models (PD Dinuc) consistently outperformed other feature classes in predicting sgRNA efficiency and were comparable to results from the combination of feature classes models in all 3 scenarios. Fusi et al. [6] and Doench et al. [7] showed that the regression model outperformed classification model using their dataset [4, 7]. However, we observed that the regression model and the classification model yielded comparable performance in both [10] and [11] datasets. The combination feature prediction model from the regression model (Comb Feature) exhibited larger AUC than both azimuth and sgRNA scorer (*p*<0.001 for all pairwise AUC comparisons using DeLong’s test [28]), but no difference using Spearman rank correlation coefficient for Comb Feature versus azimuth (*p*=0.88 from Fisher’s *Z*-transformation test [29, 30]) as shown in Additional file 1: Table S9. The results from random forest and boosted regression were presented in Additional file 1: Tables S10, S11 and S12 (randomforest) and Additional file 1: Tables S13, S14 and S15 (gbm). These results were comparable to the results from elastic net.

Following [6, 7], we also included the results from leave-one-gene out prediction framework to obtain a generalization of our prediction model to new genes in Additional file 1 (Section 5 and Tables S19 and S20). The conclusion remained the same, i.e., Comb Feature yielded the largest AUC and PD Dinuc followed closely. Additional results including performance evaluation using 30 bp sequence [6, 7] instead of 40 bp sequence were presented in Additional file 1: Tables S16, S17 and S18. The results indicated that the performance of the prediction models were comparable regardless whether a 40 bp or 30 bp sequence was used.

We created an R package predictSGRNA implementing the proposed predictive algorithm based on position-dependent dinucleotide model, available at http://www.ams.sunysb.edu/~pfkuan/softwares.html#predictsgrna.

## Conclusions

In this paper, we explored various aspects of nucleotide compositions including position dependent models, secondary structure and thermodynamics to gain better understanding of the nucleotide properties on CRISPR sgRNA design efficiency in a similar way as [6, 7]. Candidate feature ranking in terms of association with sgRNA effiency identified features which characterize the flexibility of the underlying DNA structure. Specifically, we found that the frequency of T and TT dinucleotide exhibited the strongest negative association with sgRNA efficiency. Packer et al. [14] illustrated that TT dinucleotide has the most rigid step and least flexible in terms of the ability to slide and shift, which could explain the decreased efficiency of sgRNA with higher abundance of TT dinucleotides. The results from the different predictive algorithms showed that across datasets, the position dependent mono-nucleotide model [5] achieved good operating characteristics while the prediction algorithm trained on position dependent dinucleotide model offered additional improvement in terms on AUC. The advantage of position dependent dinucleotide model in predicting sgRNA efficiency was also observed in [6, 7].

One factor that may guide improvement of future predictive algorithms is chromatin structure. Chromatin accessibility (packed vs unpacked) has been shown to be the major determinant of genome-wide binding of dCas9-sgRNA in [16]. Examples of epigenetic marks which are implicated in chromatin remodeling and accessibility include DNase I hypersensitive sites, transcription factor binding, DNA methylation and histone modification. Future work will include integrating both the nucleotide composition features and chromatin structures as features in the predictive model to characterize the binding efficiency of sgRNA.

In this study, we used datasets of size 3141 and achieved AUC of > 0.8. Prior efforts to improve the efficiency of RNAi design utilized high-throughput functional testing of the efficacy of different RNAi sequences to generate large (2182) [31] and very large datasets (∼250000) [32]. These large datasets in turn were used to develop improved prediction algorithms using machine-learning approaches similar to those used here [33, 34]. It is generally accepted that the first large test set (2182) was very useful for improving RNAi design, there is still uncertainty regarding the utility of examining very large datasets [34]. Part of the unresolved issues are the degree to which different prediction algorithms are dependent upon the vector used for shRNA expression [35] as well as the sequence context in the genome outside of the immediate target [36]. Therefore, as more CRISPR/Cas9 screens datasets are becoming available, we anticipate that the specificity of sgRNA efficacy prediction can be further improved by considering the vector-dependent level of expression of the sgRNA.

## Additional file

Additional file 1 Supplementary Information. The pdf document that contains all supplementary notes, figures and tables. Figures S1-S2 plot the top 10 most informative features ranked by BIC and variable importance scores, respectively. Tables S1-S3 contain the results from randomforest in binary outcome model. Tables S4-S6 contain the results from gbm in binary outcome model. Tables S7-S9 contain the results from elastic net in continuous outcome model. Tables S10-S12 contain the results from randomforest in continuous outcome model. Tables S13-S15 contain the results from gbm in continuous outcome model. Tables S16-S18 contain the results comparing 30bp and 40bp sequences. Tables S19-S20 contain the results from leave-one-gene out prediction. (PDF 151 kb)

## Abbreviations

AUC: Area under the receiver operating characteristic curve
Align: Optimal pairwise alignment between spacer target and scaffold
BIC: Bayesian information criterion
CRISPR: Clustered regularly interspaced short palindromic repeats
Freq: Frequencies of mono and dinucleotides
LSL: Length of a potential stem-loop
MEF: Minimum energy folding
mESC: Mouse embryonic stem cell
PAM: Protospacer adjacent motif
PD Dinuc: Position-dependent dinucleotide
PD Mono: Position-dependent mono-nucleotide
PhyChem: Physiochemical properties of [17]
PseKNC: Pseudo k-tuple nucleotide composition of [18]
Packer: Secondary structures of [14, 15]
sgRNA: Single guide RNA
Thermo: Thermodynamics and secondary structures of [9]
