# Supplementary material for: A systematic evaluation of nucleotide properties for CRISPR sgRNA design
Source: BMC Bioinformatics. 2017 Jun 6;18:297. doi: 10.1186/s12859-017-1697-6 (PMC5461693; doi:10.1186/s12859-017-1697-6)
Supplement: Additional file 1 — Supplementary Information. The pdf document that contains all supplementary notes, figures and tables. Figures S1-S2 plot the top 10 most informative features ranked by BIC and variable importance scores, respectively. Tables S1-S3 contain the results from randomforest in binary outcome model. Tables S4-S6 contain the results from gbm in binary outcome model. Tables S7-S9 contain the results from elastic net in continuous outcome model. Tables S10-S12 contain the results from randomforest in continuous outcome model. Tables S13-S15 contain the results from gbm in continuous outcome model. Tables S16-S18 contain the results comparing 30bp and 40bp sequences. Tables S19-S20 contain the results from leave-one-gene out prediction. (PDF 151 kb) [file 12859_2017_1697_MOESM1_ESM.pdf]

RESEARCH

Supplementary Information: A systematic evaluation of nucleotide properties for CRISPR sgRNA design

Pei Fen Kuan[1*], Scott Powers[2], Shuyao He[1], Kaiqiao Li[1], Xiaoyu Zhao[2] and Bo Huang[3]

[*]Correspondence: peifen.kuan@stonybrook.edu
[1]Department of Applied Mathematics and Statistics, Stony Brook University, Nicolls Road, Stony Brook, USA
Full list of author information is available at the end of the article

Candidate feature ranking

Figures S1 and S2 displayed the candidate feature ranking by the Bayesian Information Criterion (BIC) and variable importance score from the random forest prediction algorithm, respectively. The top ranked feature based on both criteria across the three datasets was the 16-th feature from PseKNC model. This feature is a function of TT dinucleotide frequency.

Results from other prediction algorithms

We also compared the results using the random forest and boosted regression to construct the predictive model. Random forest [1] was implemented in the R package `randomForest`, whereas the boosted regression based on extensions to AdaBoost [2] and gradient boosted machine [3] was implemented in the R package `gbm`. The results were shown in Tables S1, S2, S3 (`randomforest`) and Tables S4, S5, S6 (`gbm`). These results were comparable to the results from elastic net in the main text.

Results from regression model

The regression model were constructed based on (1) the average log2 fold change (12 cell doublings vs initial seeding states) of HL-60 and KBM-7 cell lines for [4] data and (2) the average log2 fold change (mESC vs plasmid control) of replicate 1 and replicate 2 of mouse ESC JM8 cell lines for [5] data. We compared the performance of the sequence properties in prediction in terms of AUC, Youden index, sensitivity, specificity; as well as Pearson correlation coefficient, Spearman rank correlation coefficient and mean squared error on the test data. The results based on elastic net were presented in Tables S7, S8 and S9. [6, 7] showed that the regression model outperformed classification model using their dataset [7]. However, we observed that the regression model and classification model yielded comparable performance in both [4] and [5] datasets. The combination feature prediction model from regression model (Comb Feature) exhibited larger AUC than both `azimuth` and `sgRNA scorer` ($p < 0.001$ for all pairwise AUC comparisons using DeLong's test [8]); but no difference using Spearman rank correlation coefficient for Comb Feature versus `azimuth` ($p = 0.88$ from Fisher's Z-transformation test [9, 10]) in Table S9. We also included the results from random forest and boosted regression. The results were shown in Tables S10, S11, S12 (`randomforest`) and Tables S13, S14, S15 (`gbm`). These results were comparable to the results from elastic net.

Results comparing 40 bp and 30 bp sequence

In this section, we compared the performance of our prediction model of the 40 bp sequence encompassing 5' flanking (10 bp) , spacer target and 3' flanking (NGG + 7 bp) region defined by [11] against 30 bp sequence (5' flanking (4 bp), spacer target and 3' flanking (NGG + 3 bp) region) as in [12, 6, 7]. Since Thermo, Packer, PhyChem, PseKNC and Align were computed for the spacer target sequence only, thus these properties were identical regardless whether 40 bp or 30 bp sequence was used, we reported the results for position dependent mono and dinucleotides in Tables S16, S17 and S18. The results indicated that the performance of the prediction models were comparable regardless whether a 40 bp or 30 bp sequence was used.

Results from leave-one-gene out prediction

Following [6, 7], we also included the results from leave-one-gene out prediction framework to obtain a generalization of our prediction model to new genes. Table S19 displayed the results from leave-one-gene out in dataset 1 (58 ribosomal genes). Table S20 displayed the results from leave-one-gene out in [4] dataset (dataset 1+ dataset 2) (220 genes).

Funding
This work was supported in part by NIH grant U01CA168409 to S.P. The funding body had no role in the design, collection, analysis or interpretation of this study.

Competing interests
The authors declare that they have no competing interests.

Author's contributions
P.K. conceived and designed the study. P.K., S.H. and K.L. carried out analyses and wrote the software. P.K., S.P., S.H., K.L., X.Z. and B.H. wrote the paper. P.K., S.P., X.Z. and B.H. critically read the manuscript and contributed to the discussion of the whole work. All authors read and approved of the final version of the manuscript.

Author details
[1]Department of Applied Mathematics and Statistics, Stony Brook University, Nicolls Road, Stony Brook, USA. [2]Department of Pathology, Stony Brook University, Nicolls Road, Stony Brook, USA. [3]Oncology Business Unit, Pfizer Inc., Eastern Point Rd, Groton, USA.

References

1. Breiman, L.: Random forests. Journal of Machine Learning **45**(1), 5–32 (2001)
2. Freund, Y., Schapire, R.: A short introduction to boosting. Journal-Japanese Society For Artificial Intelligence **14**(771-780), 1612 (1999)
3. Friedman, J.H.: Greedy function approximation: a gradient boosting machine. Annals of statistics, 1189–1232 (2001)
4. Wang, T., Wei, J., Sabatini, D., Lander, E.: Genetic screens in human cells using the CRISPR-Cas9 system. Nature **343**, 80–84 (2014)
5. Koike-Yusa, H., Li, Y., Tan, E., Mdel, C.V.-H., Yusa, K.: Genome-wide recessive genetic screening in mammalian cells with a lentiviral CRISPR-guide rna library. Nature Biotechnology **32**(3), 267–273 (2014)
6. Fusi, N., Smith, I., Doench, J., Listgarten, J.: In silico predictive modeling of crispr/cas9 guide efficiency. bioRxiv (2015)
7. Doench, J., Fusi, N., Sullender, M., Hegde, M., Vaimberg, E., Donovan, K., Smith, I., Tothova, Z., Wilen, C., Orchard, R., Virgin, H., Listgarten, J., Root, D.: Optimized sgrna design to maximize activity and minimize off-target effects of CRISPR-Cas9. Nature Biotechnology **34**(2), 184–191 (2016)
8. DeLong, E., DeLong, D., Clarke-Pearson, D.: Comparing the areas under two or more correlated receiver operating characteristic curves: a nonparametric approach. Biometrics **44**(837-845) (1988)
9. Fisher, R.: On the "probable error" of a coefficient of correlation deduced from a small sample. Metron (3-32) (1921)
10. Myers, L., Sirois, M.: Spearman correlation coefficients, differences between. Encyclopedia of Statistical Sciences **12** (2006)
11. Xu, H., Xiao, T., Chen, C., Li, W., Meyer, C., Wu, Q., Wu, D., Cong, L., Zhang, F., Liu, J., Brown, M., Liu, S.: Sequence determinants of improved CRISPR sgRNA design. Genome Research **25**, 1147–1157 (2015)

12. Doench, J., Hartenian, E., Graham, D., Tothova, Z., Hegde, M., Smith, I., Sullender, M., Ebert, B., Xavier, R., Root, D.: Rational design of highly active sgrnas for CRISPR-Cas9–mediated gene inactivation. Nature Biotechnology **32**(12), 1262–1267 (2014)

Figures

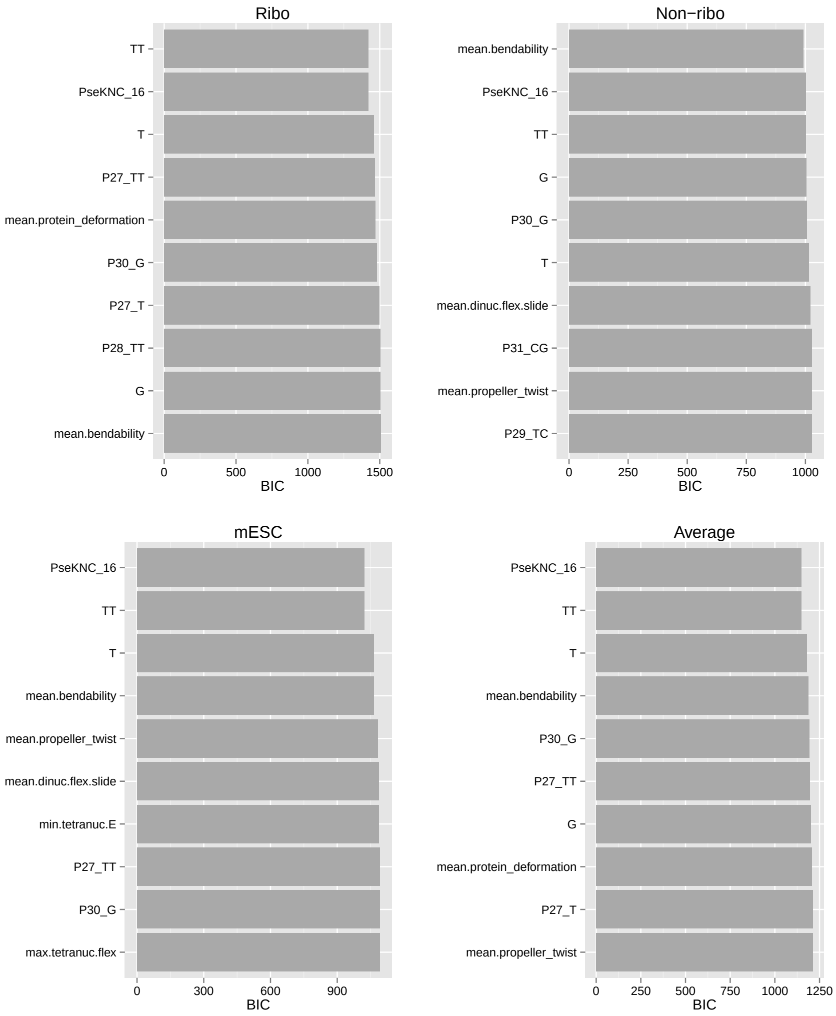

Figure S1 Top 10 most informative features ranked by BIC by dataset. The last panel is the ranking by average BIC aggregating the three datasets.

Tables

Table S1 AUC, Youden index (J), Sensitivity (Se) and Specificity (Sp) from the 3-way cross validation within dataset 1 (ribosomal genes) using random forest implemented in R package `randomForest`. Comb Feature: PD Mono+PD Dinuc+PseKNC. We reported the average performance from the 3-way cross validation over 10 iterations of random sampling.

Feature class	AUC	J	Se	Sp
PD Mono	0.824	0.528	0.806	0.723
PD Dinuc	0.843	0.545	0.825	0.720
Freq	0.743	0.394	0.781	0.614
Align	0.648	0.239	0.861	0.378
Thermo	0.577	0.146	0.718	0.428
Packer	0.634	0.236	0.597	0.639
PhyChem	0.716	0.338	0.615	0.722
PseKNC	0.728	0.380	0.768	0.612
Comb Feature	0.863	0.594	0.793	0.801

Table S2 AUC, Youden index (J), Sensitivity (Se) and Specificity (Sp) from intra-platform comparison (training set: ribosomal genes, test set: non-ribosomal genes) using random forest implemented in R package `randomForest`. Comb Feature: PD Mono+PD Dinuc+Thermo+Packer.

Feature class	AUC	J	Se	Sp
PD Mono	0.797	0.492	0.724	0.768
PD Dinuc	0.783	0.452	0.781	0.671
Freq	0.668	0.279	0.541	0.738
Align	0.611	0.171	0.918	0.253
Thermo	0.554	0.085	0.866	0.219
Packer	0.624	0.178	0.469	0.709
PhyChem	0.641	0.225	0.516	0.709
PseKNC	0.645	0.227	0.586	0.641
Comb Feature	0.804	0.468	0.823	0.646

Table S3 AUC, Youden index (J), Sensitivity (Se) and Specificity (Sp) from inter-platform comparison (training set: ribosomal and non-ribosomal genes, test set: mESC essential genes) using random forest implemented in R package `randomForest`. Comb Feature: PD Mono+PD Dinuc+Freq.

Feature class	AUC	J	Se	Sp
PD Mono	0.807	0.484	0.753	0.731
PD Dinuc	0.830	0.548	0.864	0.684
Freq	0.697	0.303	0.790	0.513
Align	0.536	0.076	0.623	0.453
Thermo	0.568	0.127	0.516	0.611
Packer	0.657	0.239	0.696	0.543
PhyChem	0.703	0.311	0.769	0.543
PseKNC	0.712	0.334	0.573	0.761
Comb Feature	0.843	0.566	0.780	0.786

Table S4 AUC, Youden index (J), Sensitivity (Se) and Specificity (Sp) from the 3-way cross validation within dataset 1 (ribosomal genes) using boosted regression implemented in R package `gbm`. Comb Feature: PD Mono+PD Dinuc+Thermo+PhyChem. We reported the average performance from the 3-way cross validation over 10 iterations of random sampling.

Feature class	AUC	J	Se	Sp
PD Mono	0.820	0.523	0.832	0.691
PD Dinuc	0.827	0.507	0.768	0.738
Freq	0.749	0.411	0.729	0.682
Align	0.642	0.254	0.825	0.429
Thermo	0.607	0.197	0.586	0.611
Packer	0.625	0.205	0.712	0.493
PhyChem	0.730	0.351	0.652	0.699
PseKNC	0.736	0.396	0.727	0.669
Comb Feature	0.860	0.578	0.781	0.797

Table S5 AUC, Youden index (J), Sensitivity (Se) and Specificity (Sp) from intra-platform comparison (training set: ribosomal genes, test set: non-ribosomal genes) using boosted regression implemented in R package `gbm`. Comb Feature: PD Mono+PD Dinuc+Freq+Align+Thermo+Packer.

Feature class	AUC	J	Se	Sp
PD Mono	0.784	0.446	0.754	0.692
PD Dinuc	0.788	0.460	0.827	0.633
Freq	0.656	0.251	0.766	0.485
Align	0.618	0.193	0.835	0.359
Thermo	0.564	0.115	0.317	0.797
Packer	0.638	0.213	0.513	0.700
PhyChem	0.636	0.212	0.617	0.595
PseKNC	0.638	0.208	0.744	0.464
Comb Feature	0.812	0.493	0.784	0.709

Table S6 AUC, Youden index (J), Sensitivity (Se) and Specificity (Sp) from inter-platform comparison (training set: ribosomal and non-ribosomal genes, test set: mESC essential genes) using boosted regression implemented in R package `gbm`. Comb Feature: PD Mono+PD Dinuc+Freq+Thermo+Packer.

Feature class	AUC	J	Se	Sp
PD Mono	0.798	0.479	0.748	0.731
PD Dinuc	0.827	0.528	0.801	0.726
Freq	0.721	0.328	0.849	0.479
Align	0.568	0.144	0.477	0.667
Thermo	0.623	0.215	0.651	0.564
Packer	0.667	0.241	0.793	0.449
PhyChem	0.710	0.331	0.669	0.662
PseKNC	0.717	0.347	0.684	0.662
Comb Feature	0.849	0.586	0.855	0.731

Table S7 AUC, Youden index (J), Sensitivity (Se), Specificity (Sp), Pearson's correlation coefficient (r) , Spearman's rank correlation coefficient (s) and Mean squared error (Mse) from the 3-way cross validation within dataset 1 (ribosomal genes) using elastic net implemented in R package `glmnet`. Comb Feature A (maximum s): PD Mono+PD Dinuc +Thermo+Packer+PhyChem+PseKNC. Comb Feature B (maximum r): PD Mono+PD Dinuc +Thermo+Packer+PhyChem+PseKNC. Comb Feature C (minimum Mse): PD Mono+PD Dinuc +Freq+Align+Thermo+Packer+PseKNC. Comb Feature D (maximum AUC): PD Mono+PD Dinuc +Thermo+PseKNC. We reported the average performance from the 3-way cross validation over 10 iterations of random sampling.

Feature class	AUC	J	Se	Sp	r	s	Mse
PD Mono	0.828	0.553	0.843	0.711	0.559	0.548	1.56
PD Dinuc	0.857	0.595	0.861	0.734	0.616	0.599	1.44
Freq	0.777	0.433	0.616	0.818	0.487	0.479	1.70
Align	0.603	0.185	0.808	0.376	0.196	0.184	2.18
Thermo	0.565	0.142	0.737	0.405	0.128	0.118	2.19
Packer	0.600	0.179	0.545	0.634	0.203	0.203	2.16
PhyChem	0.731	0.380	0.746	0.634	0.418	0.409	1.85
PseKNC	0.728	0.376	0.635	0.741	0.403	0.387	1.89
Comb Feature A	0.878	0.622	0.836	0.785	0.644	0.628	1.37
Comb Feature B	0.878	0.622	0.836	0.785	0.644	0.628	1.37
Comb Feature C	0.875	0.614	0.837	0.777	0.640	0.624	1.36
Comb Feature D	0.879	0.636	0.866	0.770	0.643	0.627	1.38

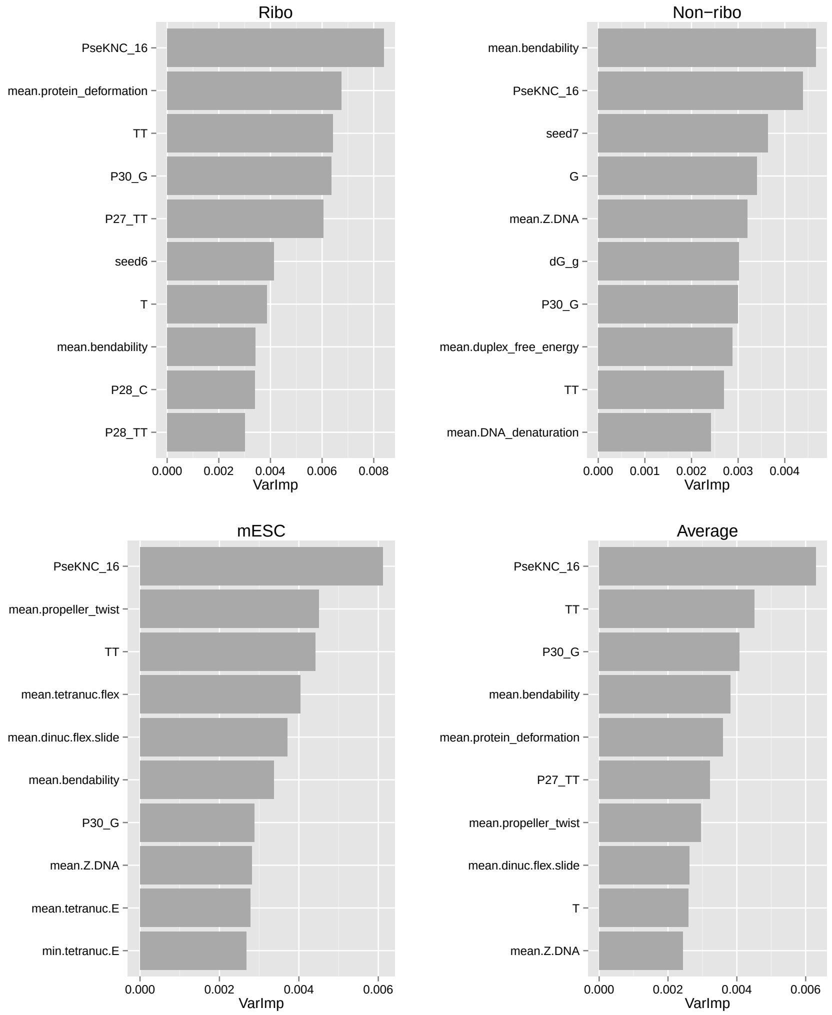

Figure S2 Top 10 most informative features ranked by unscaled permutation based variable importance scores by dataset. The last panel is the ranking by average unscaled permutation based variable importance scores aggregating the three datasets.

Table S8 AUC, Youden index (J), Sensitivity (Se), Specificity (Sp), Pearson's correlation coefficient (r) , Spearman's rank correlation coefficient (s) and Mean squared error (Mse) from intra-platform comparison (training set: ribosomal genes, test set: non-ribosomal genes) using elastic net implemented in R package `glmnet`. Comb Feature A (maximum s): PD Mono+PD Dinuc +Freq+Thermo+Packer. Comb Feature B (maximum r): PD Mono+PD Dinuc +Freq+Thermo+Packer. Comb Feature C (minimum Mse): PD Mono+PD Dinuc +Freq+Thermo+Packer+PseKNC. Comb Feature D (maximum AUC): PD Mono+PD Dinuc +Freq+Thermo+Packer.

Feature class	AUC	J	Se	Sp	r	s	Mse
PD Mono	0.780	0.432	0.697	0.734	0.459	0.436	1.36
PD Dinuc	0.799	0.492	0.787	0.705	0.480	0.429	1.30
Freq	0.713	0.351	0.790	0.561	0.351	0.310	1.51
Align	0.579	0.166	0.879	0.287	0.142	0.105	1.70
Thermo	0.596	0.172	0.535	0.637	0.147	0.129	1.72
Packer	0.641	0.231	0.510	0.722	0.210	0.174	1.62
PhyChem	0.661	0.249	0.599	0.650	0.260	0.206	1.61
PseKNC	0.649	0.241	0.642	0.599	0.249	0.191	1.59
Comb Feature A	0.816	0.536	0.832	0.705	0.516	0.466	1.25
Comb Feature B	0.816	0.536	0.832	0.705	0.516	0.466	1.25
Comb Feature C	0.814	0.541	0.849	0.692	0.515	0.463	1.25
Comb Feature D	0.816	0.536	0.832	0.705	0.516	0.466	1.25

Table S9 AUC, Youden index (J), Sensitivity (Se), Specificity (Sp), Pearson's correlation coefficient (r) , Spearman's rank correlation coefficient (s) and Mean squared error (Mse) from inter-platform comparison (training set: ribosomal and non-ribosomal genes, test set: mESC essential genes) using elastic net implemented in R package `glmnet`. Comb Feature A (maximum s): PD Mono+PD Dinuc +Align+Thermo+PhyChem. Comb Feature B (maximum r): PD Mono+PD Dinuc +Theromo+Packer+PhyChem. Comb Feature C (minimum Mse): PD Mono+Thermo+Packer. Comb Feature D (maximum AUC): PD Mono+PD Dinuc +Freq+PhyChem. Mse for azimuth and sgRNA Scorer are not computed due to scale difference.

Feature class	AUC	J	Se	Sp	r	s	Mse
PD Mono	0.798	0.497	0.783	0.714	0.395	0.300	4.38
PD Dinuc	0.831	0.530	0.773	0.756	0.492	0.374	4.21
Freq	0.750	0.381	0.714	0.667	0.328	0.223	4.87
Align	0.579	0.156	0.498	0.658	0.093	0.071	5.49
Thermo	0.639	0.253	0.778	0.474	0.155	0.043	5.04
Packer	0.661	0.242	0.580	0.662	0.157	0.071	4.90
PhyChem	0.719	0.344	0.694	0.650	0.268	0.170	4.87
PseKNC	0.734	0.364	0.736	0.628	0.282	0.149	4.78
Comb Feature A	0.843	0.553	0.843	0.709	0.506	0.383	4.08
Comb Feature B	0.844	0.561	0.808	0.752	0.506	0.382	4.09
Comb Feature C	0.815	0.507	0.717	0.791	0.439	0.336	3.71
Comb Feature D	0.847	0.564	0.799	0.765	0.503	0.372	4.19
azimuth	0.795	0.463	0.857	0.607	0.468	0.388	NA
sgRNA Scorer	0.669	0.288	0.548	0.739	0.195	0.128	NA

Table S10 AUC, Youden index (J), Sensitivity (Se), Specificity (Sp), Pearson's correlation coefficient (r) , Spearman's rank correlation coefficient (s) and Mean squared error (Mse) from the 3-way cross validation within dataset 1 (ribosomal genes) using random forest implemented in R package `randomForest`. Comb Feature A (maximum s): PD Mono+PD Dinuc +Freq+Thermo. Comb Feature B (maximum r): PD Mono+PD Dinuc +Freq+Thermo. Comb Feature C (minimum Mse): PD Mono+PD Dinuc +Freq. Comb Feature D (maximum AUC): PD Mono+PD Dinuc +Freq+Thermo. We reported the average performance from the 3-way cross validation over 10 iterations of random sampling.

Feature class	AUC	J	Se	Sp	r	s	Mse
PD Mono	0.825	0.550	0.790	0.761	0.561	0.543	1.577
PD Dinuc	0.836	0.532	0.756	0.776	0.573	0.542	1.539
Freq	0.748	0.392	0.771	0.622	0.436	0.423	1.811
Align	0.641	0.256	0.698	0.557	0.270	0.226	2.079
Thermo	0.587	0.167	0.736	0.432	0.163	0.148	2.235
Packer	0.640	0.232	0.521	0.711	0.244	0.245	2.100
PhyChem	0.720	0.347	0.635	0.711	0.406	0.398	1.869
PseKNC	0.726	0.375	0.751	0.624	0.409	0.400	1.858
Comb Feature A	0.864	0.602	0.864	0.738	0.637	0.613	1.439
Comb Feature B	0.864	0.602	0.864	0.738	0.637	0.613	1.439
Comb Feature C	0.860	0.595	0.782	0.813	0.630	0.604	1.436
Comb Feature D	0.864	0.602	0.864	0.738	0.637	0.613	1.439

Table S11 AUC, Youden index (J), Sensitivity (Se), Specificity (Sp), Pearson's correlation coefficient (r) , Spearman's rank correlation coefficient (s) and Mean squared error (Mse) from intra-platform comparison (training set: ribosomal genes, test set: non-ribosomal genes) using random forest implemented in R package `randomForest`. Comb Feature A (maximum s): PD Mono+PD Dinuc +Packer. Comb Feature B (maximum r): PD Mono+PD Dinuc +Packer. Comb Feature C (minimum Mse): PD Mono+PD Dinuc +Freq+Align+Packer. Comb Feature D (maximum AUC): PD Mono+PD Dinuc +Packer.

Feature class	AUC	J	Se	Sp	r	s	Mse
PD Mono	0.790	0.465	0.832	0.633	0.494	0.444	1.272
PD Dinuc	0.773	0.433	0.791	0.641	0.444	0.381	1.357
Freq	0.672	0.270	0.726	0.544	0.269	0.233	1.610
Align	0.599	0.182	0.908	0.274	0.210	0.157	1.688
Thermo	0.572	0.122	0.291	0.831	0.122	0.116	1.817
Packer	0.632	0.202	0.434	0.768	0.215	0.190	1.638
PhyChem	0.638	0.217	0.575	0.641	0.208	0.179	1.757
PseKNC	0.653	0.234	0.639	0.595	0.240	0.212	1.637
Comb Feature A	0.804	0.478	0.744	0.734	0.519	0.459	1.238
Comb Feature B	0.804	0.478	0.744	0.734	0.519	0.459	1.238
Comb Feature C	0.801	0.479	0.791	0.688	0.518	0.454	1.234
Comb Feature D	0.804	0.478	0.744	0.734	0.519	0.459	1.238

Table S12 AUC, Youden index (J), Sensitivity (Se), Specificity (Sp), Pearson's correlation coefficient (r) , Spearman's rank correlation coefficient (s) and Mean squared error (Mse) from inter-platform comparison (training set: ribosomal and non-ribosomal genes, test set: mESC essential genes) using random forest implemented in R package `randomForest`. Comb Feature A (maximum s): PD Mono+PD Dinuc +Freq+PhyChem. Comb Feature B (maximum r): PD Mono+PD Dinuc +Freq+Packer. Comb Feature C (minimum Mse): PD Mono+PD Dinuc +Freq+PhyChem. Comb Feature D (maximum AUC): PD Mono+PD Dinuc +Freq+Packer.

Feature class	AUC	J	Se	Sp	r	s	Mse
PD Mono	0.784	0.456	0.789	0.667	0.428	0.315	4.332
PD Dinuc	0.823	0.517	0.769	0.748	0.486	0.351	4.236
Freq	0.701	0.311	0.863	0.449	0.277	0.184	4.743
Align	0.540	0.096	0.280	0.816	0.065	0.062	7.180
Thermo	0.537	0.104	0.839	0.265	0.148	0.124	4.929
Packer	0.663	0.282	0.680	0.603	0.208	0.135	4.801
PhyChem	0.704	0.342	0.705	0.637	0.275	0.195	4.799
PseKNC	0.714	0.339	0.617	0.722	0.247	0.148	5.118
Comb Feature A	0.830	0.559	0.849	0.709	0.502	0.378	4.093
Comb Feature B	0.838	0.576	0.863	0.714	0.509	0.363	4.104
Comb Feature C	0.830	0.559	0.849	0.709	0.502	0.378	4.093
Comb Feature D	0.838	0.576	0.863	0.714	0.509	0.363	4.104

Table S13 AUC, Youden index (J), Sensitivity (Se), Specificity (Sp), Pearson's correlation coefficient (r) , Spearman's rank correlation coefficient (s) and Mean squared error (Mse) from the 3-way cross validation within dataset 1 (ribosomal genes) using boosted regression implemented in R package `gbm`. Comb Feature A (maximum s): PD Mono+PD Dinuc +Thermo+PhyChem. Comb Feature B (maximum r): PD Mono+PD Dinuc +Thermo+PhyChem. Comb Feature C (minimum Mse): PD Mono+PD Dinuc +Freq+Thermo+PhyChem. Comb Feature D (maximum AUC): PD Mono+PD Dinuc +Thermo. We reported the average performance from the 3-way cross validation over 10 iterations of random sampling.

Feature class	AUC	J	Se	Sp	r	s	Mse
PD Mono	0.822	0.531	0.843	0.688	0.543	0.534	1.618
PD Dinuc	0.844	0.530	0.743	0.787	0.579	0.565	1.609
Freq	0.749	0.409	0.732	0.677	0.446	0.432	1.787
Align	0.643	0.256	0.808	0.449	0.298	0.243	2.057
Thermo	0.598	0.187	0.705	0.483	0.179	0.165	2.176
Packer	0.630	0.210	0.646	0.564	0.243	0.234	2.101
PhyChem	0.732	0.353	0.598	0.755	0.430	0.419	1.818
PseKNC	0.738	0.394	0.759	0.635	0.424	0.411	1.835
Comb Feature A	0.858	0.583	0.778	0.805	0.616	0.602	1.454
Comb Feature B	0.858	0.583	0.778	0.805	0.616	0.602	1.454
Comb Feature C	0.857	0.595	0.795	0.800	0.613	0.600	1.452
Comb Feature D	0.859	0.603	0.810	0.793	0.604	0.590	1.521

Table S14 AUC, Youden index (J), Sensitivity (Se), Specificity (Sp), Pearson's correlation coefficient (r) , Spearman's rank correlation coefficient (s) and Mean squared error (Mse) from intra-platform comparison (training set: ribosomal genes, test set: non-ribosomal genes) using boosted regression implemented in R package `gbm`. Comb Feature A (maximum s): PD Mono+PD Dinuc +Packer. Comb Feature B (maximum r): PD Mono+PD Dinuc +Align+Packer. Comb Feature C (minimum Mse): PD Mono+PD Dinuc +Freq+Align+Packer. Comb Feature D (maximum AUC): PD Mono+PD Dinuc +Packer.

Feature class	AUC	J	Se	Sp	r	s	Mse
PD Mono	0.771	0.416	0.724	0.692	0.447	0.424	1.350
PD Dinuc	0.757	0.387	0.729	0.658	0.424	0.364	1.403
Freq	0.657	0.256	0.733	0.523	0.250	0.206	1.629
Align	0.600	0.195	0.875	0.321	0.231	0.145	1.638
Thermo	0.568	0.119	0.520	0.599	0.142	0.142	1.703
Packer	0.638	0.217	0.432	0.785	0.226	0.196	1.615
PhyChem	0.644	0.230	0.656	0.574	0.232	0.196	1.673
PseKNC	0.643	0.207	0.823	0.384	0.236	0.185	1.641
Comb Feature A	0.795	0.476	0.779	0.696	0.488	0.446	1.291
Comb Feature B	0.793	0.459	0.890	0.570	0.492	0.442	1.282
Comb Feature C	0.792	0.474	0.870	0.603	0.489	0.438	1.280
Comb Feature D	0.795	0.476	0.779	0.696	0.488	0.446	1.291

Table S15 AUC, Youden index (J), Sensitivity (Se), Specificity (Sp), Pearson's correlation coefficient (r) , Spearman's rank correlation coefficient (s) and Mean squared error (Mse) from inter-platform comparison (training set: ribosomal and non-ribosomal genes, test set: mESC essential genes) using boosted regression implemented in R package `gbm`. Comb Feature A (maximum s): PD Mono+PD Dinuc +Freq+Thermo+PhyChem. Comb Feature B (maximum r): PD Mono+PD Dinuc +Freq+Thermo+PhyChem. Comb Feature C (minimum Mse): PD Mono+PD Dinuc +Freq+Thermo+Packer. Comb Feature D (maximum AUC): PD Mono+PD Dinuc +Freq+Thermo+Packer.

Feature class	AUC	J	Se	Sp	r	s	Mse
PD Mono	0.788	0.470	0.748	0.722	0.384	0.292	4.442
PD Dinuc	0.810	0.477	0.853	0.624	0.468	0.334	4.354
Freq	0.724	0.345	0.759	0.585	0.281	0.165	4.714
Align	0.571	0.138	0.480	0.658	0.098	0.087	7.007
Thermo	0.626	0.233	0.780	0.453	0.183	0.059	5.067
Packer	0.665	0.247	0.863	0.385	0.204	0.111	4.830
PhyChem	0.705	0.321	0.748	0.573	0.265	0.156	4.818
PseKNC	0.714	0.338	0.616	0.722	0.233	0.132	5.303
Comb Feature A	0.826	0.538	0.846	0.692	0.479	0.338	4.186
Comb Feature B	0.826	0.538	0.846	0.692	0.479	0.338	4.186
Comb Feature C	0.829	0.533	0.755	0.778	0.476	0.327	4.146
Comb Feature D	0.829	0.533	0.755	0.778	0.476	0.327	4.146

Table S16 AUC, Youden index (J), Sensitivity (Se) and Specificity (Sp) from the 3-way cross validation within dataset 1 (ribosomal genes) using elastic net for 40bp and 30bp sequences.

Feature class	AUC (40bp)	AUC (30bp)
PD Mono	0.826	0.831
PD Dinuc	0.858	0.864

Table S17 AUC, Youden index (J), Sensitivity (Se) and Specificity (Sp) from intra-platform comparison (training set: ribosomal genes, test set: non-ribosomal genes) using elastic net for 40bp and 30bp sequences.

Feature class	AUC (40bp)	AUC (30bp)
PD Mono	0.785	0.783
PD Dinuc	0.792	0.792

Table S18 AUC, Youden index (J), Sensitivity (Se) and Specificity (Sp) from inter-platform comparison (training set: ribosomal and non-ribosomal genes from, test set: mESC essential genes) using elastic net for 40bp and 30bp sequences.

Feature class	AUC (40bp)	AUC (30bp)
PD Mono	0.797	0.795
PD Dinuc	0.832	0.830

Table S19 AUC, Youden index (J), Sensitivity (Se) and Specificity (Sp) from leave-one-gene out prediction within dataset 1 (ribosomal genes) using elastic net classification.

Feature class	AUC	J	Se	Sp
PD Mono	0.839	0.543	0.840	0.703
PD Dinuc	0.873	0.604	0.796	0.808
Freq	0.780	0.430	0.692	0.737
Align	0.611	0.177	0.599	0.578
Thermo	0.520	0.068	0.922	0.146
Packer	0.603	0.177	0.572	0.605
PhyChem	0.728	0.357	0.791	0.566
PseKNC	0.738	0.380	0.715	0.664
Comb Feature	0.883	0.623	0.844	0.779

Table S20 AUC, Youden index (J), Sensitivity (Se) and Specificity (Sp) from leave-one-gene out prediction in [4] data (i.e., dataset 1+dataset 2) using elastic net classification.

Feature class	AUC	J	Se	Sp
PD Mono	0.822	0.510	0.830	0.680
PD Dinuc	0.848	0.567	0.828	0.738
Freq	0.771	0.423	0.700	0.724
Align	0.595	0.151	0.650	0.501
Thermo	0.584	0.145	0.622	0.524
Packer	0.623	0.184	0.647	0.537
PhyChem	0.715	0.342	0.765	0.577
PseKNC	0.720	0.342	0.752	0.590
Comb Feature	0.857	0.581	0.863	0.719
